# Zinc Dies

**Published:** 1900-03

**Authors:** Oliver Perry Wolfe

**Affiliations:** Canton, Mass.


ARTICLE V.

### ZINC DIES.

OLIVER PERRY WOLFE, D. M. D., CANTON, MASS.

I have a few words to say upon that branch of our profession which relates to the making of zinc dies on which to strike up a gold plate.

It seems to me that the various works on prosthetic dentistry deal to lightly with this particular subject, and as we all realize that the ultimate success or failure of a case depends almost entirely on the model upon which it is made, I think that a description of the method by which I make my dies might be interesting.

While in college we were taught to run the model in plaster, building it up an inch or so, and sandpaper the sides until they were smooth. Then chalk was applied and an impression taken in sand. We were told that the sand should be just wet enough, not too moist, and too dry. In fact it should have just the right "feel." Then we were told to pour it with zinc. If the model had undercuts which we could not overcome by drawing the model at an angle, we were to fill them in with chalk and cut them out in the zinc model while it was hot.

How many burned fingers, how many ruffled tempers and how many ill-fitting plates this has caused I could not attempt to state, but suffice to say that many dentists to day are putting vulcanite or celluloid in mouths, where gold could and should be used, and for what reason? They dread the undercuts.

Nearly a year ago a patient came to my office and requested me to make a gold plate. The mouth was in good condition and everything seemed favorable for a good-fitting denture. The impression was duly taken and handed to my assistant, with instruction to pour it and take an impression in sand. An hour later he came to me and said that he could not get a good impression, owing to the exceedingly deep undercuts.

I took the model and after several trials, the last time filling the undercuts with chalk, I succeeded in getting the model away without breaking the sand. But this was not satisfactory. All semblance to the original model was lost, and I knew that a great deal of cutting must be done on the zinc to bring it down to the proper shape. It then occurred to me to try an experiment, the details of which I am about to give.

NOVEL METHOD OF MAKING A ZINC DIE.

I covered the model with a thin coating of vaseline and set it on the slab, just as we do when about to pack the sand around it. Then I mixed sand and plaster in the following proportions: Sand, three parts; plaster, one part; just thin enough to run easily, and poured it over the model, tapping the slab to insure a perfect adaptation to all parts. As it began to set, I built it up to thickness of an inch above the face of the model, and then allowed it to set. I then put it over a low flame until perfectly dry. Then with a knife I cut a narrow groove from the center in front to the center behind, nearly down to the model. Then with one hand on each side I broke it apart, took out the model, and put the pieces together again, the fractures being so clean that when wired together the crack could not be detected.

This gave me a perfect impression, and we then proceeded to pour it with zinc. There was just enough plaster mixed with the sand to keep it firmly together, and as it was perfectly dry, I had no trouble with steam.

While my assistant poured the zinc, I gently tapped the impression and the slab on which it rested, exactly as we do when pouring a plaster model.

The result exceeded my expectations, for it gave a perfect die, entirely devoid of air bubbles or other imperfections.

The plaster and sand impression did not seem to suffer in the slightest, and might have been used again had we so desired.

The counter die of lead was made in the usual manner, and of course had to be pried off the zinc model.

The plate which I struck up on this die was the best that had ever left my office. Since then I have made several on this plan, and in each case have met with flattering success.

Should any of my brother practitioners try this method, or if they can suggest a better way, I should be pleased to hear from them.—*Items of Interest.*

